# Implementation of a new cost efficacy method for blood irradiation using a non dedicated device

**DOI:** 10.1186/1756-9966-30-7

**Published:** 2011-01-12

**Authors:** Paola Pinnarò, Antonella Soriani, Daniela D'Alessio, Carolina Giordano, Maria Laura Foddai, Valentina Pinzi, Lidia Strigari

**Affiliations:** 1 Radiotherapy Department, Regina Elena National Cancer Institute, Rome, Italy; 2 Laboratory of Medical Physics and Expert Systems, Regina Elena National Cancer Institute, Rome, Italy; 3 Transfusion Department, Regina Elena National Cancer Institute, Rome, Italy

## Abstract

**Objectives:**

To implement a new cost efficacy internal Service for blood component irradiation, we carried out specific procedures and quality assurance reports using the linear accelerators (LINACs) of the Regina Elena Institute (IRE) Radiotherapy Department instead of a dedicated device.

**Methods:**

The technical aspects, quality assurance and regulatory requirements of the internal procedure to set up a local irradiated blood bank have been defined. The LINACs of the IRE Radiotherapy Department were used to deliver a mean dose of 32 Gy and dose accuracy was checked with gafchromic film. The overall time/cost of this procedure was compared with the previous procedure, out-sourcing the irradiation of blood components.

**Results:**

A total of 1996 blood component units were internally irradiated in the first year. Moreover, reducing the overall procedure time by a third. Overall cost/bag of external and internal procedures was approx. 66 € and 11 €, respectively. Thus the average saving of cost/bag was higher than 80%. The use of gafchromic films in all irradiated blood component bags allowed the accuracy of the dose delivered to blood to be checked.

**Conclusions:**

By utilizing LINACs installed in the Radiotherapy Department it is possible to provide an internal blood component irradiation service, capitalizing on internal resources without any inconvenience/discomfort to patients undergoing radiotherapy and satisfying governmental regulatory requirements. The internal irradiation procedures has proven to be safe and feasible, and along with the significant cost/time reduction suggests that it is more advantageous than external procedures.

## Introduction

Blood component irradiation is the only proven method of preventing a risk of transfusion-associated graft versus host disease (TA-GVHD) [1].

This immunologic reaction of engrafted lymphocytes against the host system is intense and proves fatal in about 90% of affected patients [2].

The irradiation of blood components inhibits lymphocyte function avoiding damage to the platelets and other blood fractions. Moreover, it renders T-lymphocytes incapable of replication without affecting the function of RBCs, granulocytes, and platelets. The irradiation can be performed using a dedicated blood irradiation device based on Cesium-137 [3] or a Cobalt-60 source, or else an X-ray device.

Each radiation machine has specific constructive design and energy which determine the time and methods of blood bag irradiation within an appropriate dose range.

Studies on the radiosensitivity of T cells to X-rays and to gamma rays have shown that a minimum dose of 25 Gy is necessary to prevent TA-GVHD [3-6]. Moreover, the dose must not exceed 50 Gy in order to avoid harming the function or decreasing the life span of red blood cells, platelets or granulocytes [3,7-10].

Although there have not been any reported cases of TA-GVHD following platelet transfusion alone, the same irradiation method is applied due to the fact that platelets are also contaminated with a small number of lymphocytes [3].

Red cells may be irradiated at any time up to 14 days after collection and thereafter stored for a further 14 days from irradiation. Where the patient is at particular risk from hyperkalaemia, it is recommended that red cells be transfused within 24 hours of irradiation. Platelets can be irradiated at any stage in their five-day storage and can thereafter be stored up to their normal shelf life of five days after collection. Granulocytes for all recipients must be irradiated as soon as possible after production due to the reduction in functionality of the WBC during storage time, and should thereafter be transfused with minimum delay [3].

The Regina Elena (IRE) is a major National Cancer Research Institute providing oncology services and encompassing eight Surgery Departments, two Medical Oncology Departments, one Haematology Department, one Transfusion Department and one Radiotherapy Department, as well as a variety of support services. In our Institute, the number of patients at GVHD risk who might require transfusions of irradiated components is relevant (accounting for more than 2000 bags per year) and blood irradiation represents an important, although ancillary, service to complete a primary mission of caring.

Due to the fact that there is no dedicated device at the IRE, the blood component bags have previously been out-sourced for irradiation. In order to reduce the cost, the logistic problems and the time of procedure, the implementation of a proven cost/time saving blood component irradiation procedure based on internal resources has been required of the Radiotherapy and Medical Physics Departments by the IRE Administration.

Several publications have focused on the technical aspects of the irradiation process itself [3], but relatively little attention has been paid to the economical and managerial details [11]. The main aim is to report the experience of IRE in the implementation of an internal blood irradiation program using a conventional linear accelerator (LINAC), as an alternative to out-source services. The secondary aim is to compare the overall time and costs of both internal and external procurement of blood components.

## Materials and methods

In our Institute, patients at risk for TA-GVHD for whom irradiated blood or products are requested include those with: haematological malignancy or solid tumor (Glioblastoma, Neuroblastoma, Rhabdomyosarcoma); Hodgkin's disease treated with ablative chemo/radiotherapy; non-Hodgkin's lymphoma; acute leukemia (ANLL and ALL), recipients of peripheral blood or bone marrow stem cell transplants (Allogeneic, Autologous), diseases treated with Fludaribine and other potent purine analogues, diseases treated with Cladribine (deoxycoformycin). Until June 2009 blood components were sent out to external Transfusion Departments with conventional Cs-137 sources, with significant expense of time/cost due to transport safety of the blood component bags.

Due to the distance between IRE and the external Departments and the traffic of a big city, the overall time of the external procedure varies from 2 to 3 hours including delivery time, acceptance and the irradiation duration (mean 2.5 h). This procedure requires the availability of a car, a driver and an operator of the centre of Transfusion Department to deliver the irradiated blood components. Moreover, a further payment of 38 euro (€)/irradiation for each bag was established by the Healthy Ministry.

In the first half of 2009, in our Institute, the request for irradiated blood bags increased by 40% compared to 2008, leading to an increase of logistical problems and costs.

So the opportunity to use one of the three LINACs available in the Radiation Oncology Department of IRE has been considered on the condition that this does not affect the number of patients or prolong the waiting time of treatment in any way. The three LINACs are matched to be permanently set for the same output calibration, flatness and symmetry, which ensure the same dose distribution delivery based on the identical machine input data.

A procedure based on rigorous modus operandi, careful dosimetric checks and quality assurance programs have been implemented and a cost-benefit evaluation has been conducted.

In particular, the procedure time and the number of irradiated blood components were registered on a form. The number and qualification of personnel involved in both procedures (external and internal) have been identified and their work time has been computed and a comparison of the two procedures has been carried out.

### Design of a blood irradiation container and set-up

To facilitate and standardize the blood component irradiation using a linear accelerator, a blood irradiator box was designed and made of Polymethylmethacrylate (PMMA).

**Figure 1:**
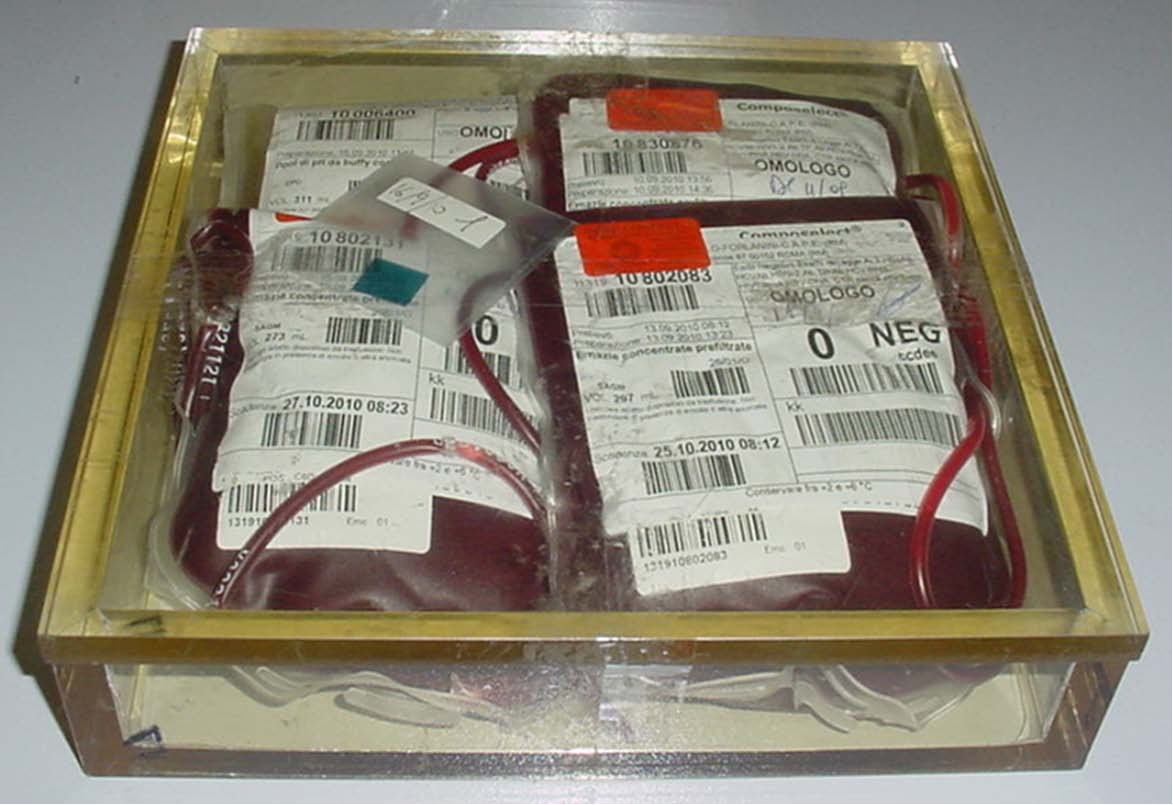
**box filled with blood bags**.

The PMMA box of 24 × 24 × 5.5 cm^3 ^is large enough to accommodate a maximum of 4 bags of packed RBCs or 10 bags of platelets (Figure 1). The thickness of the box walls and the top layer is 1 cm, while the bottom layer is 0.5 cm, to guarantee an appropriate build-up of 6 MV photon.

**Figure 2:**
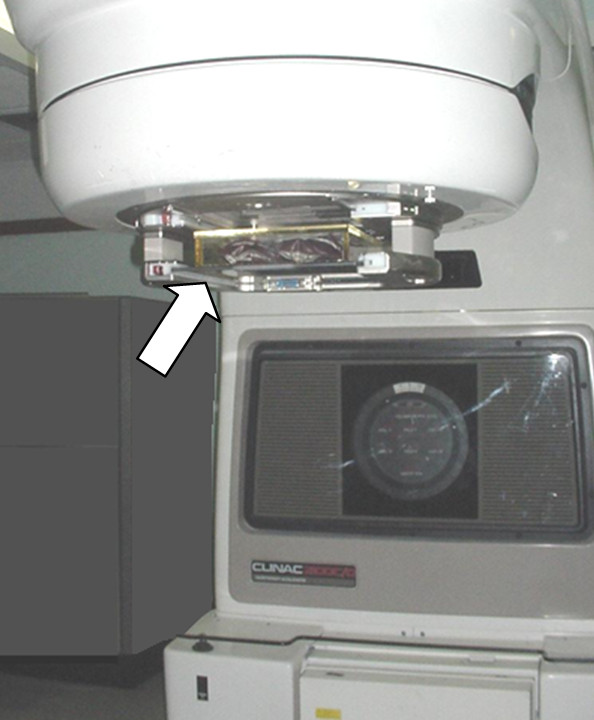
**Box fixed at the head of the LINAC (see arrow)**.

The box fits into the block tray at the head of the linear accelerator (Varian 2100C/D, Palo Alto CA). The distance from the source and the surface of the box (SSD) is fixed (about 60 cm) and only one 6 MV direct field of 40 × 40 cm^2 ^at the isocenter was used with a gantry angle of 0° (Figure 2).

This one-field technique facilitates a reproducible administration of the dose to blood units and considerably reduces the irradiation time.

The CT scan of the box filled with four blood bags was performed for a treatment planning study. A Pinnacle 8.0 m Treatment Planning system, i.e. TPS, (Philips Medical Systems, Madison, WI) was used to calculate the three-dimensional dose distribution of bags. The prescribed dose was at least 25 Gy avoiding hot spots over 45 Gy. The calculated total Monitor Units were 922 with a rate of 600 Monitor Units/min, resulting in a dose-rate of 19.5 Gy/min.

The blood bags were delineated on the CT images, the dose distribution of a 6 MV photon beam (gantry 0°) and the dose volume histograms (DVHs) of the inner of box and bags were calculated. Using the distribution calculation generated by TPS, the dose distribution within the box is sufficiently homogeneous and does not depend on the number of bags placed in the box to be irradiated. Based on these multiple calculations and measurements performed during the implementation phase, the individual units of RBCs or platelets were sufficiently irradiated - also considering different setups (e.g. number of bags placed in each box). This allows us to confirm the correct choice of the setup configuration (LINAC and box into the block tray) in order to guarantee the minimum and maximum dose to blood components.

The plan was sent to the Varis Record and Verify (R&V) system to guarantee the highest level of safety regarding the set-up and dose delivery. The overall delivery time was about 3 min/box.

The time out of refrigeration of the blood component units was limited to 15 minutes, amply within the maximum admissible time for these kind of blood components i.e. 45 minutes.

### Procedure of irradiation components

The procedure for blood component irradiation was established as follows.

The irradiation of blood components is performed at the Radiotherapy Department on the request of the Transfusion Service. The personnel must: (a) compile the request for irradiation (one for each box) to include the sequencial number, the date, the label with the code (CDM), one for each unit to be irradiated; (b) place the blood component units to be irradiated in the box (i.e. up to 4 bags of blood or 10 of platelets), positioning them to fill any gaps and placing each CDM in order to be easily visible from the box top for final checking (see Figure 1); (c) place one dosimeter (i.e. gafchromic film) in each box, then fill in the accompaning form with the irradiation date and the number of box used; (d) transport the hermetically seal boxes to the Radiotherapy Department and wait for the completion of the irradiation procedure.

The Radiotherapy Technician must verify that the CDMs in the box correspond to those on the irradiation request, start dose delivery; check the colour of the dosimeter, fill in the form with the delivered monitor units and give a copy to the Transfusion Department Technician.

Finally, the Medical Physicist must collect the dosimeters and check the dose delivered.

Each day before beginning the treatments the accuracy of the dose delivery is checked using the Double Check Instrument (Model 7200 Victoreen), according to the LINAC quality assurance programme.

### Gafchromic Calibration

Before dosimetric verification, an MD-V2-55 gafchromic calibration curve was obtained for different dose levels ranging from 0.01 to 50 Gy, by using LINAC calibrated according to IAEA TRS 398 protocol [12]. Film pieces of 1.5 × 1.5 cm^2 ^were cut for the gafchromic calibration and irradiated in a solid water phantom (30 × 30 × 30 cm^3^), which had been placed on the LINAC couch at SSD = 90 cm and SAD = 100 cm. The set-up was 6 MV photon beam (gantry angle: 0°, field: 10 × 10 cm^2^). The dose was delivered with one of the three LINACs (Clinac 2100/CD Varian).

The gafchromic films were read by an Epson 10000 × L Scanner with a maximum spatial resolution of 1600 × 3200 dpi. All acquisition data were obtained by positioning the MD-V2-55 gafchromic film at the centre of the scan region, according to literature [13,14]. Films were scanned using Picodose film dosimetry software (Tecnologie Avanzate, Italy) and the images were saved into file format (.sun). The MD-V2-55 gafchromic showed a linear trend from 0.01 to 50 Gy in accordance with the technical specifications.

The gafchromic films for dosimetric verification are 1.5 × 1.5 cm^2 ^and are routinely placed in the blood component box during irradiation.

## Results

### Planning, commissioning and dosimetry

**Figure 3:**
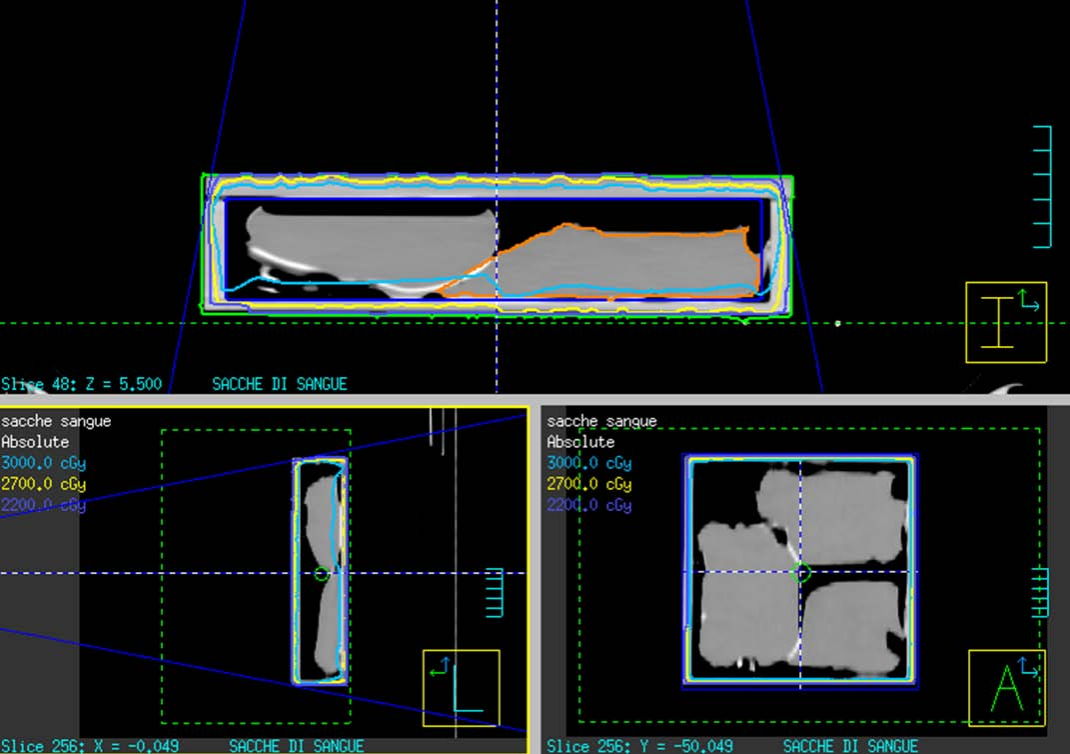
**Isodose distribution calculated with Pinnacle TPS within the box**.

In the implementation phase the isodose distribution was determined within the filled box using Pinnacle TPS (Figure 3). Using the one field technique, the minimum and the maximum dose of blood component were 27 Gy and 35 Gy, respectively.

More than 500 pieces of gafchromic films (at least one for each box) were used for dose verification choosing a particular reference point close on the box top for this purpose.

The average measured value with gafchromic films was 31.4 ± 1.8 Gy in agreement with that expected, i.e. 32 Gy.

### Irradiated blood components

The average number of platelets and blood bags were 118 and 48, respectively per month. The total number of blood components irradiated at IRE in the first year with the internal procedures was 1996.

### Procedure time

Assuming that each box contains 5 bags on average, we estimated that the "work time" of personnel involved is 29.2 versus 12.2 minutes for external and internal procedures, respectively, for each bag irradiated (Table 1 and 2).

**Table 1:** Average external and internal procedure time for each bag irradiated

	External procedure time (minutes)	Internal procedure time (minutes)
Contracted Driver	**9**	**-**

Technician (Radiotherapy Dep.)	**-**	**0.5**

Dosimetric verifier (Physicist)	**-**	**0.5**

Technician (Transfusion Dep.) (§)	**29.2**	**12.2**

(§) more details regarding time and procedure are reported in Table 2.

**Table 2:** Procedure and time (average and range, when appropriate) for each irradiated box (5 bags) carried out by personnel of the Transfusion Department

Procedure	External procedure time (minutes)	Internal procedure time (minutes)
Call for arrangements	**15**	**0**

Select unit components	**5**	**5**

Preparation phase (+ fax)	**6 (range: 5-7)**	**6 (range: 5-7)**

Contracted driver, delivery and collection of irradiated units	**15**	**0**

Preparation of blood components	**10**	**10**

Time total (from leaving to returning to the transfusion department)	**75 (range: 60-90)**	**30 (range: 20-40)**

Load procedure of blood components by the transfusion department	**20**	**10**

Total	**146 (range: 130-162)**	**61 (range: 50-72)**

### Costs

The average cost per bag includes the average cost of consumable supplies, of personnel and the depreciation of equipment.

Indirect costs for internal procedures include LINAC (100,00 €/h) and the scanner depreciation (2,00 €/h). Indirect cost for external procedures mainly include the transport of blood component bags.

Direct costs for internal procedures are mainly related to the gafchromic film. On average, direct and indirect costs are 0,23 and 0,65 € per bag, respectively.

The cost for personnel involved are; IRE technicians approx. 42 € per hour and Medical Physicist approx. 67 € per hour (data provided by the IRE Administration).

The cost of internal dosimetric verification is 1,00 €/bag.

The list of costs for external and internal procedures is reported in Table 3 per bag.

**Table 3:** Comparison of costs/bag irradiated with external and internal procedures

	COSTS for External procedures (€/bag)	COSTS for Internal procedures (€/bag)
Indirect cost (§)	8	0,65

Direct cost (°)	-	0,23

Technician (Transfusion Dep.) (°°)	20,44	8,54

Technician (Radiotherapy Dep.) (°°)	-	0,63

Dosimetric verification (°°)	-	1,00

Cost for one irradiation to be corresponded to External Institute	38	-

**Total cost for blood bag**	**66,44**	**11,05**

Note: (§) assuming also the cost of LINAC depreciation (100 €/h), the scanner depreciation (2 €/h); (°) including the cost of gafchromic films; (°°) see Table 1 and 2 for the time.

The cost of the implementation of the internal procedure was 144,24 € and included the cost of the box and the treatment planning study.

One thousand nine hundred and ninety six blood components were irradiated internally in the first year, so the overall savings to IFO was about € 110.558,44. All the blood component bags were transfused.

## Discussion

The procedure was developed, verified and has since been successfully implemented in the Transfusion, Medical Physics and Radiotherapy Departments, irradiating about two thousand blood components internally in the first year.

The one-field irradiation procedure is much more easy to perform and time saving compared to other techniques reported in literature and based on LINAC [11-13].

There is no allowance for set-up error and the entire dose delivery procedure lasts only 3 minutes/box. The blood components are irradiated at the request of the Transfusion Department. The procedure is no longer carried out soley according to daily necessity but also on a regular weekly basis and stored for up to two weeks.

The IRE procedure delivering a mean dose of 32 Gy (range: 27-35 Gy) is in accordance with the Italian Decree [14] and International Recommendations [3].

The gafchromic film, inserted into each box, is a visual reminder that the blood components have been irradiated, and the data analysis guarantees that the intended dose matches with that delivered. In fact, the gafchromic films serve multiple purposes: 1) to avoid a erroneous (no/duplicated) irradiation of the same box when multiple irradiations are programmed in the same session; 2) to measure the dose delivered to a particular reference point, close to the box top; 3) to implement a quality control programme of blood irradiation. In our experience, the use of gafchromic film confirms the accuracy of measured dose in agreement with other Authors [13,15,16]. Of relevance based on TPS calculations, checking the dose at the reference point we can confirm the dose distribution at any point in the box. Moreover, the numer of bags within the box makes no significant changes to the dose distribution, as confirmed by multiple calculations and measurements performed during the implementation phase.

Finally, the forms reporting the blood component bag code and the value of delivered dose are filed in both the Radiotherapy and Transfusion Departments, while the irradiated gafchromic films are stored in the Medical Physics Department.

After an initial cost of about 144 €, the total cost for blood component bags for external and internal procedures is very different (about 66 vs 11 €/bag, respectively). The internal procedure avoids logistic problems as the blood components do not have to be transported out of the IRE.

The overall savings of IFO was about € 110.558 due to the irradiation of 1996 blood components in the first year, without affecting in any way the scheduled treatments in the Radiotherapy Depatment. The overall saving was about 83% per bag. In conclusion, we assume that the efficacy of both procedures is the same, the minimum and the maximum dose being in the range recommended by international guideline, thus the cost-efficacy study corresponds to the cost analysis. However, the cost and the time per bag are lower in the internal than in the external procedure. Thus, the internal procedure is preferable when an Institute has LINACs for patient radiotherapy, while the external procedure could be useful over the week-end (i.e. when the regular activity of the Radiotherapy Department is closed).

## Conclusion

By utilizing LINACs installed in the Radiotherapy Department it is possible to provide an internal blood component irradiation service, capitalizing on internal resources without any inconvenience/discomfort to patients undergoing radiotherapy. The development and organization of such an irradiation program requires rigorous modus operandi and careful dosimetric checks, to ensure the quality of the irradiated components and to satisfy governmental regulatory requirements. In our procedure the delivered dose accuracy has been assessed by gafchromic film in a PMMA box. This and a very simplified irradiation set-up provide a fast and reliable way to guarantee that the delivered dose is in accordance with international guidelines.

In conclusion, the internal irradiation procedures has proven to be safe and feasible, and along with the significant cost/time reduction suggests that it is more advantageous than external procedures in Istitutes/Hospitals without dedicated devices.

## Competing interests

The authors declare that they have no competing interests.

## Authors' contributions

PP and AS made conception and designed. PP, MLF and AS coordinated the study. VP, DD, CG, MLF collected data. LS, PP, DD, CG, MLF and AS analyzed data, carried out data interpretation. LS, AS and PP participated in drafting of manuscript. All authors read and approved the final manuscript.
